# Data on the ultrastructural characteristics of *Paenibacillus polymyxa* isolates and biocontrol efficacy of *P. polymyxa* ShX301

**DOI:** 10.1016/j.dib.2018.09.058

**Published:** 2018-09-26

**Authors:** Fan Zhang, Xiao-Lin Li, Shui-Jin Zhu, Mohammad Reza Ojaghian, Jing-Ze Zhang

**Affiliations:** Ministry of Agriculture, Key Lab of Molecular Biology of Crop Pathogens and Insects, College of Agriculture and Biotechnology, Zhejiang University, Hangzhou 310058, China

## Abstract

We present the data corresponding to the ultrastructural characteristics of *Paenibacillus polymyxa* isolates and control efficacy of *P. polymyxa* ShX301 for controlling Verticillium wilt of cotton, isolated in experimental fields at the Sanyuan Agricultural Experiment Station of North-West Agriculture and Forestry University, Sanyuan county, Shaanxi province, China. Ultrastructural characteristics of *P. polymyxa* isolates made using technique of transmission electron microscopy. A strain ShX301 has a broad-spectrum antifungal activity against *V. dahliae* and other plant pathogens and has been used for *in vitro* experiments for controlling this disease in greenhouse, "Biocontrol potential of *Paenibacillus polymyxa* against *Verticillium dahliae* infecting cotton plants" [1].

**Specifications table**

**Table:** 

Subject area	*Biology*
More specific subject area	*Microbiology, Microscopy*
Type of data	*Transmission electron microscopy (TEM) images, Tables*
How data was acquired	*TEM following an optimized cell preparation protocol*
	*Statistical analysis explained in the text of this article*
Data format	*Analyzed*
Experimental factors	*Bacterial cells were grown in solid media for TEM observation, inoculation and experiment condition (explained in the text of this article)*
Experimental features	*Ultrastructural characteristics were made using technique of transmission electron microscopy*
Data source location	Paenibacillus polymyxa isolates were isolated from the experimental fields at the *Sanyuan* Agricultural *Experiment Station of* North-West Agriculture and Forestry University, *Sanyuan county*, Shaanxi province, China.
Data accessibility	*Data incorporated within this article and the sequences of Paenibacillus polymyxa isolates has been deposited in GenBank under the accession number*KX458008*,*KX458009*and*KX458010.

**Value of the data**

• Our data provide the evidence that hints the three-layered spore coat is possibly a common feature in genus *Peanibacillus*.
• Biocontrol assay showed that *P. polymyxa* strain ShX301 has great potential using as biocontrol bacterium for controlling Verticillium wilt of cotton.
• The data can be used for general analysis of bacterial identification and screening of biocontrol strains.

## 1 Data

**Fig. 1:**
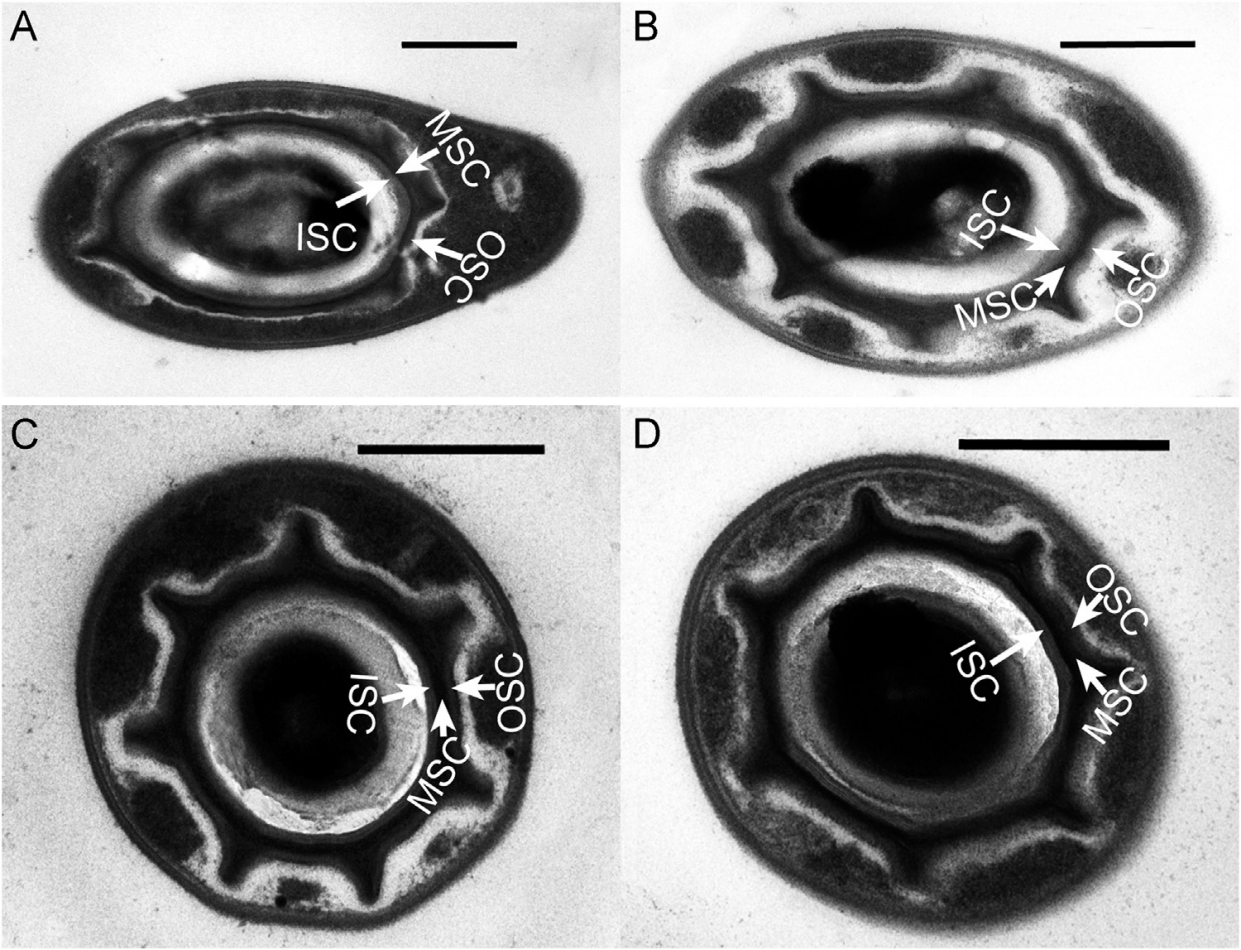
Transmission electron micrographs of endospores of *Paenibacillus polymyxa* grown on specific spore-forming medium at 30 °C for 48 h. A. Strain Hb1. B. Strain Hb6. C. Strain ShX302. D. Strain ShX303. ISC: inner spore coat. OSC: outer spore coat. MSC: middle spore coat. Bar = 0.5 µm.

Sporulation process of *Paenibacillus polymyxa* strain ShX301 was described [1], which was similar to that described in *P. motobuensis* by Iida et al. [2]. While other four strains (Hb1, Hb6, ShX302 and ShX303) of *P. polymyxa* also shared the same characteristics with strain ShX301. The mature spores in the sporangia all had the three-layered spore coats in the four strains (Fig. 1).

Inoculation tests showed that inoculation by strain ShX301 reduced disease incidence and severity (1). The raw information related to disease incidence and severity contained in the Table 1.

**Table 1:** Inhibitory efficacy of *P. polymyxa* ShX301 against Verticillium wilt of cotton^a^.

**Disease grade**		**0**	**1**	**2**	**3**	**4**	**Disease severity (%)**	
**Treatment1 (*****V. dahliae*****+*****P. polymyxa*****ShX301)**	R1	48	2	4	6	2	15.30	13.50 ± 1.58
	R2	50	1	4	5	2	12.90	
	R3	48	3	7	3	1	12.31	
**Treatment2 (*****V. dahliae*****)**	R1	3	20	14	12	13	57.63	53.83 ± 1.67
	R2	6	21	12	13	11	50.29	
	R3	5	21	10	14	13	53.57	
**Ck1 (*****P. polymyxa*****ShX301)**	R1	61	0	0	0	0	0.0	0.0
	R2	61	0	0	0	0	0.0	
	R3	63	0	0	0	0	0.0	
**Ck2 (sterile water)**	R1	62	0	0	0	0	0.0	0.0
	R2	63	0	0	0	0	0.0	
	R3	61	0	0	0	0	0.0	

ΔDisease severity was assessed for each plant on a 0 to 4 rating scale according to the percentage of foliage affected by acropetal chlorosis, necrosis, wilt, and/or defoliation (0 = healthy plant, 1 = 1 to 33%, 2 = 34 to 66%, 3 = 67 to 99%, 4 = dead plant)as described by Bejaranoalcazar et al. [3].

a The disease assessment was carried out 45 days after planting for each plant on a 0 to 4 rating scale (0 = healthy plant, 1 = 1–33%, 2 = 34–66%, 3 = 67–99%, 4 = dead plant). Disease severity (%) = Σ (disease ratings × number of plants)/(maximum rating value × Total number of plants) × 100. R: repetition.

## 2 Experimental design, materials and methods

For endospore observation, bacterial strains were grown on specific spore-forming medium (10 g beef extract, 2 g yeast extract, 0.04 g manganese II sulphate monohydrate, 25 g agar, pH 7.2) for two days at 25 °C [4]. Ultrastructural characteristics were observed using a JEM-1010 transmission electron microscope (JEOL USA Inc., Peabody, MA, USA).

The seeds of a susceptible cotton (*Gossypium hirsutum* cv. Ejing-1) were used. The disease assessment was carried out 45 days after planting. Disease severity was assessed for each plant on a 0 to 4 rating scale [3]. Please see the publication "Biocontrol potential of *Paenibacillus polymyxa* against *Verticillium dahliae* infecting cotton plants." (Zhang et al. [1]) for the details of Experimental design, materials and methods.
