# Supplementary material for: Data on the ultrastructural characteristics of Paenibacillus polymyxa isolates and biocontrol efficacy of P. polymyxa ShX301
Source: Data Brief. 2018 Sep 26;21:259–62. doi: 10.1016/j.dib.2018.09.058 (PMC6197323; doi:10.1016/j.dib.2018.09.058)
Supplement: Supplementary file 1 — Supplementary material [file mmc1.docx]

**CONFLICT OF INTEREST STATEMENT**

We confirm that the manuscript has been read and approved by all named authors and that there are no other persons who satisfied the criteria for authorship but are not listed. We further confirm that no conflict of interest exists.
