# Supplementary material for: Data on the ultrastructural characteristics of Paenibacillus polymyxa isolates and biocontrol efficacy of P. polymyxa ShX301
Source: Data Brief. 2018 Sep 26;21:259–62. doi: 10.1016/j.dib.2018.09.058 (PMC6197323; doi:10.1016/j.dib.2018.09.058)
Supplement: Supplementary file 2 — Supplementary material [file mmc2.docx]

**Supplementary Figure 1**


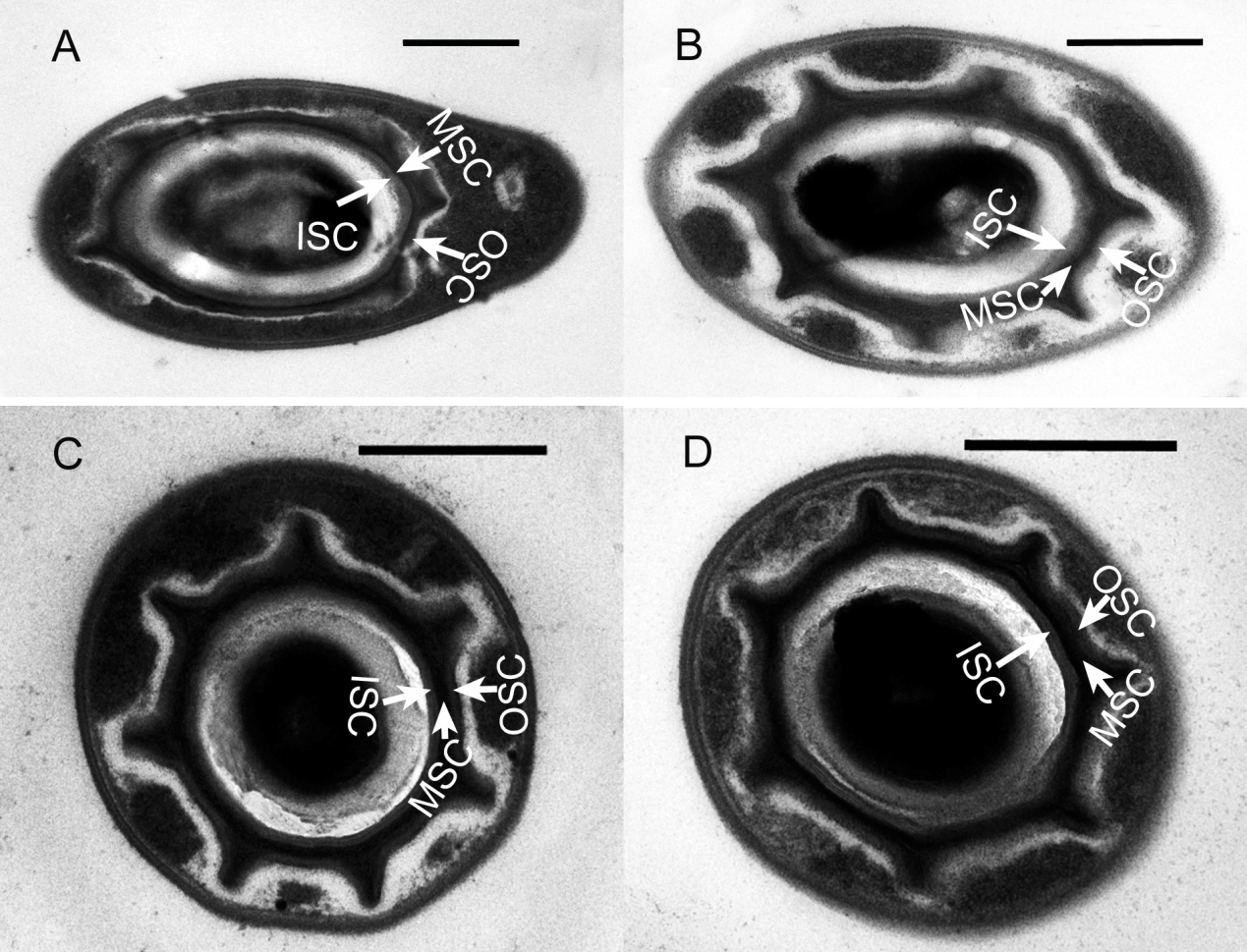
Fig. 1. Transmission electron micrographs of endospores of *Paenibacillus polymyxa* grown on specific spore-forming medium at 30 °C for 48 h. A. Strain Hb1. B. Strain Hb6. C. Strain ShX302. D. Strain ShX303. ISC: inner spore coat. OSC: outer spore coat. MSC: middle spore coat. Bar = 0.5µm.
