# Supplementary material for: Data on the ultrastructural characteristics of Paenibacillus polymyxa isolates and biocontrol efficacy of P. polymyxa ShX301
Source: Data Brief. 2018 Sep 26;21:259–62. doi: 10.1016/j.dib.2018.09.058 (PMC6197323; doi:10.1016/j.dib.2018.09.058)
Supplement: Supplementary file 3 — Supplementary material [file mmc3.doc]

**Supplementary** **Table 1.** Inhibitory efficacy of *P. polymyxa* ShX301 against Verticillium wilt of cotton*.

| **Disease grade** |  | **0** | **1** | **2** | **3** | **4** | **Disease severity (%)** | |
| --- | --- | --- | --- | --- | --- | --- | --- | --- |
| **Treatment1**  **(*V. dahliae* + *P. polymyxa* ShX301)** | R1  R2  R3 | 48  50  48 | 2  1  3 | 4  4  7 | 6  5  3 | 2  2  1 | 15.30  12.90  12.31 | 13.50±1.58 |
| **Treatment2**  **(*V. dahliae*)** | R1  R2  R3 | 3  6  5 | 20  21  21 | 14  12  10 | 12  13  14 | 13  11  13 | 57.63  50.29  53.57 | 53.83±1.67 |
| **Ck1**  **(*P. polymyxa* ShX301)** | R1  R2  R3 | 61  61  63 | 0  0  0 | 0  0  0 | 0  0  0 | 0  0  0 | 0.0  0.0  0.0 | 0.0 |
| **Ck2**  **(sterile water)** | R1  R2  R3 | 62  63  61 | 0  0  0 | 0  0  0 | 0  0  0 | 0  0  0 | 0.0  0.0  0.0 | 0.0 |

*The disease assessment was carried out 45 days after planting for each plant on a 0 to 4 rating scale (0 = healthy plant, 1 = 1 to 33%, 2 = 34 to 66%, 3 = 67 to 99%, 4 = dead plant). Disease severity (%) = Σ (disease ratings × number of plants)/ (maximum rating value × total number of plants) × 100. R: repetition.

^△^Disease severity was assessed for each plant on a 0 to 4 rating scale according to the percentage of foliage affected by acropetal chlorosis, necrosis, wilt, and/or defoliation (0 = healthy plant, 1 = 1 to 33%, 2 = 34 to 66%, 3 = 67 to 99%, 4 = dead plant）as described by Bejaranoalcazar et al.(1995).
